# How Social Support and Parent–Child Relationships Related to LGBTQ+ College Students’ Academic Challenges During COVID-19

**DOI:** 10.3390/ijerph22030459

**Published:** 2025-03-20

**Authors:** Yuan Zhang, Miranda R. Garcia, Eva. S. Lefkowitz

**Affiliations:** 1 School of Social and Family Dynamics, Arizona State University, Tempe, AZ 85287, USA; yzha1325@asu.edu; 2 Women’s Resource Center, Student Engagement and Wellbeing, Georgia Institute of Technology, Atlanta, GA 30332, USA; miranda.garcia@studentlife.gatech.edu; 3 Department of Human Development and Family Sciences, University of Connecticut, Storrs, CT 85287, USA

**Keywords:** COVID-19, LGBTQ+ college students, academic challenges, social support, parent–child relationships

## Abstract

The COVID-19 pandemic disrupted the living arrangements of many college students in the United States, potentially impacting their academic development, which plays a critical role in their mental health. At the start of the pandemic, university closures led to an abrupt transition from face-to-face instruction to online instruction, which may have caused significant challenges for college students, particularly lesbian, gay, bisexual, transgender, queer, and others who identify as having a minority sexual orientation and/or gender identity (LGBTQ+). To identify academic challenges and associated protective factors, we examined LGBTQ+ college students’ social support from family and friends, the parent–child relationship quality, and their associations with academic challenges during the first months of the pandemic. The results of online surveys indicated that LGBTQ+ college students (*N* = 408; Mean Age = 20.4 yrs) who reported less family support and worse relationship quality with their parents perceived that academics had become relatively harder than before the pandemic. In contrast, friend support was unrelated to perceived academic challenges. These findings underscore the potentially protective role of supportive and high-quality relationships with family. The findings also provide insight into how universities could support students’ academic success during other temporary academic breaks and sudden, unplanned disruptions, such as hurricanes or other weather-related events, which is essential in promoting LGBTQ+ college students’ mental health and academic success.

## 1. Introduction

The COVID-19 pandemic disrupted the living arrangements of many college students in the United States (U.S.), potentially impacting their academic development. This disruption may have disproportionately affected lesbian, gay, bisexual, transgender, queer, and others who identify as having a minority sexual orientation and/or gender identity (LGBTQ+) [1]. Academic-related stressors are among the most dominant factors affecting college students’ mental health, a concern that was further amplified during the pandemic [2,3]. Therefore, it is essential to focus on the academic challenges students encountered during this period, particularly within vulnerable populations such as LGBTQ+ students.

At the start of the pandemic, many college students experienced a sudden and significant shift when U.S. universities closed their campuses, and most residential students had to move off-campus, often to live with family members, in an immediate and appropriate public health response [4,5]. This interruption to their education caused by the unexpected transition from traditional face-to-face to online classes may have led to college students experiencing increased difficulty in focusing on their education [6]. For example, the transition from college campuses designed to provide supportive learning communities to remote learning from less traditional learning environments may have caused challenges for college students amidst the pandemic, such as challenges in balancing studying and other life demands [7]. Many students expressed concern that they would fail to fulfill their educational goals, such as completing their coursework on time, and would encounter other professional or personal development barriers as a result of the transition [8]. Overall, the abrupt transition to remote learning during the pandemic underscored significant disruptions in college students’ academic progress, highlighting the need for targeted support to address their educational and personal challenges.

In addition, campus closures might also have led to other difficulties that prevented college students from making effective progress in their schoolwork. For example, campus closures might have limited students’ access to resources (e.g., physical libraries, computers, or free internet) [9], and students reported often having technical issues during classes, like lack of access to WiFi or difficulty finding a quiet place to work [10]. Such difficulties might have led to challenges in managing coursework and other tasks compared to pre-pandemic academic experiences. In addition to academic challenges, many college students encountered other hardships such as financial difficulties, job loss, and food insecurity [5,11,12]. Also, the first months of the pandemic disrupted many U.S. college students’ living arrangements, and many students moved out of residence halls to live with their families after the pandemic started [13]. The change in living arrangements may have added responsibilities that students do not juggle when living on campus, such as caring for younger or older family members. The changes may also have led to increased stressors, such as dealing with family illness or loss due to COVID-19, or living in more crowded spaces with less privacy and dedicated time for attending online classes, completing homework, and taking exams [5,10]. These extra burdens may have contributed to the challenges students reported in concentrating on their schoolwork or balancing their schoolwork and personal life.

LGBTQ+ students may have been at particular risk for the negative influence of campus closures because, as delineated by Minority Stress Theory, they experience unique stressors as members of a marginalized community [14,15], which might negatively impact their academic performance [5,16]. The pandemic displaced many LGBTQ+ students from potentially supportive campus environments in which students have access to greater resources and a strong sense of community to off-campus housing with potentially less supportive individuals [17]. For instance, many LGBTQ+ college students have families who do not know about their gender identity/expression and/or sexual orientation, and the minority-related stressor of concealing their identity could lead to additional stress [18,19]. In addition, even if they have disclosed their sexual orientation and/or gender identity to their families, many LGBTQ+ students also experience unsupportive environments such as parental disapproval or rejection [16], which may have contributed to additional stress during the first months of the pandemic. All of these stressors likely led to increased academic challenges during the first months of the pandemic for LGBTQ+ students. Prior research has not demonstrated an association between LGBTQ+ college students’ changes in living arrangements and perceived changes in academic performance due to the pandemic [20]. However, this prior research only considered whether the students’ living arrangements changed or not, and not their interpersonal contextual experiences. In the current study, we consider how two contextual experiences were associated with students’ academic challenges during the first months of the pandemic: perceived social support from family or friends, and the relationship quality with parents.

Social support from family and friends and relationships with parents are critical contextual factors with implications for academic performance [21]. Although we know of no work that has considered the role of family or friends in academic performance during the pandemic, evidence suggests that the parent–child relationship quality and social support from family and friends mattered for LGBTQ+ students’ mental health during the first months of the pandemic [22] and that a supportive environment is beneficial to LGBTQ+ college students’ success outside the pandemic context [23]. Thus, in this paper, we describe LGBTQ+ college students’ academic challenges during the pandemic, and we consider how perceived social support from family or friends and the quality of the relationship with parents were associated with LGBTQ+ students’ academic challenges during the first months of the pandemic. These results not only are important for understanding LGBTQ+ college students’ experiences during the pandemic but also have implications for other expected (e.g., academic break) or unexpected (e.g., closure due to weather phenomena) times when college students must leave campus and return to their parents’ homes.

Specifically, in the current study, we aimed to explore the following research questions (RQs):

**RQ1:**  How did LGBTQ+ college students perceive the pandemic’s impact on their ability to concentrate on schoolwork, manage tasks, and balance academic and personal life?
**RQ2:**  Did LGBTQ+ college students with less social support from family and friends perceive that academics had become harder due to the COVID-19 pandemic than students with more support?
**RQ3:**  Did LGBTQ+ college students with worse-quality relationships with their parents perceive that academics had become harder due to the COVID-19 pandemic than students with better-quality relationships?

## 2. Materials and Methods

### 2.1. Procedure

To recruit the sample for the larger LGBTQ+ Crew Study (see [24] for more information), we identified all of the LGBTQ+ Resource Centers at public, four-year universities in the U.S. with an enrollment of over 5000 students whose spring semester was still in session at the time of data collection using the Higher Education LGBT Support Services Map (https://www.lgbtcampus.org/find-an-lgbtq-campus-center (accessed on 1 April 2020)). We contacted 98 of these Resource Centers and requested they disseminate study information and a survey link to their LGBTQ+ undergraduate students. Students from 32 of these universities, located in all four census regions, completed an online Qualtrics survey between 29 April and 25 May 2020.

The participants first answered inclusion criteria questions to confirm their age, LGBTQ+ identity, undergraduate student status, and enrollment at a participating university. We invited students who qualified to participate in the full survey. Participation took approximately 30 min, and students received a USD 20 Amazon gift card upon survey completion. We recruited a total of 455 participants for the larger study. The current sample consisted of the 408 students who completed all individual items and at least 50% of any scales used in the analyses in the current paper. Approval for the LGBTQ+ Crew Study was obtained from the University of Connecticut Institutional Review Board.

### 2.2. Participants

The participants were 18 to 24 years old (M = 20.4, SD = 1.4) and identified as a member of the LGBTQ+ community. For gender identity, 48.8% of the sample identified as cisgender women, 20.3% cisgender men, 14.5% non-binary, 7.1% transgender men, 3.2% transgender women, 2.9% genderqueer, and 3.2% other. For sexual orientation, 37.7% identified as bisexual, 16.9% gay, 14.7% lesbian, 15.2% queer, 7.1% pansexual, 4.9% asexual, 0.7% heterosexual/straight, and 2.7% other. For ethnicity, 24.3% identified as Hispanic/Latinx (H/L). Among the non-H/L participants, 59.3% identified as White, 14.2% Asian, 4.7% Black, 2.0% North African/Middle Eastern, 1.7% American Indian/Alaskan Native, and 0.2% other.

### 2.3. Measures

*Perceived Change in Academic Challenges.* To assess the change in academic challenges due to the COVID-19 pandemic, we created three questions: “How has your ability to concentrate on schoolwork changed since the start of social distancing/self-isolation related to the COVID-19 pandemic?”; “How has your ability to successfully manage your tasks changed since the start of social distancing/self-isolation related to the COVID-19 pandemic?”; and “How has your ability to balance your schoolwork and your personal life changed since the start of social distancing/self-isolation related to the COVID-19 pandemic?”. The response options were 1 = much easier than before, 2 = somewhat easier than before, 3 = a little easier than before, 4 = has not changed, 5 = a little harder than before, 6 = somewhat harder than before, and 7 = much harder than before. We created a mean score for the perceived change in academic challenges (α = 0.90).

*Social Support*. We adapted the Norbeck Social Support Questionnaire (NSSQ) [25] to assess social support separately for family and friends. The scale consists of six questions about each target, answered on a five-point scale, from 0 = not at all to 4 = a great deal (sample question: “How much do your family/friends make you feel liked or loved?”). We created separate mean scores for family and friends (α = 0.87 for both).

*Parent–Child Relationship Quality*. We used the Parent and Adult Relationship Questionnaire (PARQ) [26] to assess the relationship quality between students and their parents. Participants who did not have a relationship with at least one parent could choose not to answer the questions and, thus, were excluded from the analytic sample. The PARQ consists of eight items for each parent, with two subscales: positive affect (four items; e.g., “How often has your mother/father acted warm or affectionate toward you?”) and negative affect (four items; e.g., “How often has your mother/father acted angry or hostile toward you?”). Participants responded separately for their mother and father using a five-point Likert scale, from 1 = never to 5 = always. We created an overall relationship quality score separately for mothers (α = 0.89) and fathers (α = 0.87) by reversing the negative-affect items. For the current analyses, we created a mean score of mothers’ and fathers’ scores as the parent–child relationship quality score. If participants reported on only one parent, we used their report for the one parent as their score.

### 2.4. Data Analysis Plan

First, to answer RQ1, we calculated descriptive statistics to describe the frequency of perceived change in LGBTQ+ college students’ academic challenges. To test RQ2 and RQ3, we first constructed regression models separately for each contextual factor, including all control variables in Step 1 and social support (friends or family) or parent–child relationship quality in Step 2, with the perceived change in academic challenges during the pandemic as the outcome. Next, we conducted one multivariate regression with all control variables entered in Step 1 and social support (friends and family) and parent–child relationship quality in Step 2, with the perceived change in academic challenges as the outcome. In all regression models, we controlled for factors identified in prior research as correlates of academic stress or performance (i.e., gender identity [1 = women; 0 = not]; transgender/gender non-conforming [1 = yes; 0 = no]; sexual orientation [1 = plurisexual/asexual; 0 = monosexual]; living with parent(s) [1 = living with at least one parent; 0 = not]; living with others [1 = living with someone other than parents; 0 = not]; out to parent(s) [1 = yes; 0 = no]; out to others [1 = out to people other than parents; 0 = not]; student’s year of school [1 = junior/senior; 0 = freshman/sophomore], and employment status [1 = employed; 0 = not]) [19,27,28,29,30,31,32].

## 3. Results

### 3.1. Results Summary

Overall, in support of RQ1, the majority of the participants perceived a substantial increase in academic challenges as a result of the pandemic. Specifically, 78.0% reported it was somewhat or much harder to concentrate on schoolwork, 69.6% reported it was somewhat or much harder to successfully manage their schoolwork, and 67.4% reported it was somewhat or much harder to balance schoolwork and their personal life than it was before the pandemic (see Table 1). The modal response for all three items was that it was much harder than before the pandemic.

Two of the three bivariate models (social support from family and parent–child relationship quality) and the multivariate linear regression model were significant (see Table 2). Overall, the multivariate model explained 8% of the variance in perceived change in academic challenges due to the pandemic.

#### 3.1.1. Social Support

As presented in Table 2, although not significant in the multivariate model, social support from family was significantly associated with students’ perceived changes in academic challenges in the bivariate model, providing partial support for RQ2. Compared to their peers with more family social support, students who reported less social support from their family perceived that academics had become relatively harder than before the pandemic. Social support from friends was not significantly associated with the perceived change in academic challenges in either bivariate or multivariate models.

#### 3.1.2. Parent–Child Relationship Quality

Parent–child relationship quality was significantly associated with students’ perceived changes in academic challenges in both the bivariate and multivariate models, in support of RQ3 (see Table 2). Compared to students who reported better-quality relationships with their parent(s), students who reported a worse relationship quality with their parents perceived that academics had become relatively harder than before the pandemic. 

## 4. Discussion

Guided by Minority Stress Theory [14,15], we examined LGBTQ+ college students’ perceived changes in academic-related challenges and their association with perceived social support from family and friends and parent–child relationship quality. Our findings indicate that during the first months of the pandemic, when universities were still in session but had closed their physical campuses, LGBTQ+ college students experienced increased academic challenges at rates comparable to or higher than those in students’ retrospective reports from summer 2020 in other published work (e.g., [33]). In the current paper, we specifically found that the majority of LGBTQ+ college students reported increased difficulties managing and concentrating on their schoolwork and balancing schoolwork with their personal life compared to before the pandemic. We cannot determine whether these challenges were due to the stress of the pandemic, the challenges of balancing school life with living (for many students) with family, or the change in living environments. Future research could examine whether students who live at home experience more academic challenges than students who live on campus. In addition, research should consider whether these challenges are heightened for LGBTQ+ college students compared to their heterosexual, cisgender peers.

Prior research did not find an association between living arrangements and academic performance for LGBTQ+ college students during the COVID-19 pandemic [20]. In the current paper, living arrangements, one of our control variables, were also not associated with academic challenges. However, we extended this research by examining associations between changes in academic challenges and interpersonal relationship factors. We found that although social support from peers was not linked to changes in academic challenges, less social support from family and worse-quality relationships with parents were associated with experiencing a larger increase in academic challenges. This finding aligns with prior research suggesting that family support was critical for LGBTQ+ students’ mental health and well-being both before [34] and during the pandemic [22,33]. Many students moved out of residence halls and in with family after campus closures, and they had limited in-person contact with others due to social distancing [24]. As a result, LGBTQ+ students may have become more reliant on family members during the pandemic. Given the established association between academic stress and mental health [2], understanding the role of family support in alleviating academic challenges is critical for promoting LGBTQ+ students’ academic success and overall well-being. Future research should similarly consider the role of family support in LGBTQ+ students’ academic achievement and experiences in other times of heightened stress or disruption, as well as during normative changes such as academic breaks when many college students return to live with family.

This study has several limitations. First, the cross-sectional data preclude causal inferences. It may be that academic challenges led to reduced family social support and relationship quality with parents, rather than the reverse. That is, students who experienced increased academic challenges may have extended this stress to their interactions with their family, leading to decreased relationship quality with family members. Second, although data collection during the early stage of the pandemic effectively captured students’ immediate experiences rather than relying on retrospective memory, it also presents a limitation. In April and May of 2020, students would not yet recognize the long-term or delayed impacts of the pandemic on their academic challenges, as changes in university structure continued for many months following. Third, we cannot determine the accuracy of students’ perceptions. Students reported on their perceived changes in academic challenges, but because we do not have data from before the start of the pandemic, we do not know whether these challenges actually increased. Fourth, the association between key predictor variables reduced the number of significant correlates in the multivariate model, so that some correlates were only significant in the bivariate models. Fifth, we included only students who identified as LGBTQ+ at 4-year public universities. Students who were still exploring their identity but did not yet feel comfortable may not have chosen to participate in the current study, and, thus, our results may be less applicable to students with less established identities. In addition, students who attended different types of universities, such as community colleges where students are more likely to live with family year-round, may have had different experiences as a result of the pandemic. Sixth, because the students were exclusively LGBTQ+, we cannot compare this population to their heterosexual/cisgender peers. Without a comparison, we cannot know that this population experienced disproportionate academic challenges. Finally, scholars have emphasized the importance of addressing the unique needs of LGBTQ+ college students within specific subgroups [35]. Despite our sample’s diversity, it was not large enough to consider differences by gender identity, sexual orientation, ethnic, or racial subgroups. It may be, for instance, that students with multiple minority statuses experienced even more academic challenges than students who were only gender minority, sexual minority, or ethnic/racial minority [36], or that social support and family relationships played a differential role for different subgroups.

Despite these limitations, this study creates opportunities for future research and provides suggestions for higher education and student-affairs practices. Researchers should consider LGBTQ+ students’ experiences during normative academic breaks. In particular, given the increased reliance on remote courses during winter and summer sessions, researchers should consider whether LGBTQ+ students experience increased academic challenges during remote learning compared to residential learning, along with the role of family and peer support and relationships in these challenges. Understanding these academic challenges is crucial as they can contribute to heightened stress levels, which, in turn, may negatively impact students’ mental health and overall well-being [2]. Future research should also consider how intersectional historically marginalized/minoritized identities such as race/ethnicity, ability, and social class may exacerbate academic challenges [36].

Prior research suggests that unexpected interruptions, such as the COVID-19 pandemic, can exacerbate existing obstacles, disproportionately affecting college enrollment and persistence for minoritized students [37]. In the current study, we similarly found that LGBTQ+ college students perceived increased academic challenges due to the pandemic. Although pandemics are rare disruptions, habitual transitions, such as winter and summer breaks, also result in disruptions to the regular routine, which might also present academic challenges for college students. These disruptions in academic routines can exacerbate stress for LGBTQ+ students, a group already vulnerable to mental health disparities due to minority stress [15,33]. For LGBTQ+ students who may not have supportive home environments, universities could offer more flexible residence hall access during planned breaks by keeping a limited number of halls open year-round or creating emergency housing options.

To further support students during academic breaks, universities should also consider developing or improving remote support systems. Providing access to mental health services during academic breaks is especially critical, as LGBTQ+ students often rely on campus counseling centers that are tailored to their needs. Without these services, the academic stress experienced during breaks may further contribute to negative mental health outcomes [33,38]. Universities should consider increasing access to mental health services during academic breaks and virtual spaces for peer mentoring, tutoring, and academic advising to ensure that LGBTQ+ students continue to have access to academic and social support networks even when off-campus. For instance, academic centers and tutoring services should be adapted for easy accessibility during summer and winter breaks for students taking remote courses. Providing technological flexibility for students to discreetly discuss academic and personal matters from less supportive households would also be beneficial.

In addition to scheduled disruptions, college students sometimes experience other sudden and unplanned disruptions. For instance, in September 2022, in advance of Hurricane Ian, all major Florida public universities and many private universities closed their campuses, unexpectedly dislocating many thousands of students [39]. Similar closures happened in North Carolina in October 2024 as a result of Hurricane Helene [40]. Our findings suggest that LGBTQ+ students may be at risk of academic challenges during such unplanned disruptions. Given that academic stress is a well-established contributor to poor mental health outcomes [3], universities must proactively support LGBTQ+ students to mitigate both the academic and emotional consequences of sudden disruptions. Although universities may not be able to provide residence hall access during such closures, they should consider the living arrangements for LGBTQ+ students who may not have safe homes to return to. In addition, much as for planned academic breaks, universities should consider access to campus resources during unplanned campus closures, which may decrease barriers to students seeking similar support to what is offered when classes are in session [40]. Further, given that perceived support from instructors is linked to students’ social integration and ability to connect with peers [41], universities should encourage instructors to respond to student feedback, grant flexibility, and be creative in their approach to supporting students during planned or unplanned disruptions to in-person classes.

Students on residential campuses often have access to affordable or free mental and physical healthcare during the semester. Further, counseling centers and other wellness resources on college campuses may offer services tailored to the LGBTQ+ community. All of these opportunities may provide students with support to decrease their academic stress and challenges. However, when LGBTQ+ students are away from campus, they may face heightened barriers to accessing mental healthcare, which can compound the stress of academic challenges and exacerbate mental health disparities [33,38]. When universities cannot provide access to remote care, they could collaborate with community-based resources and provide students with referrals to help them more easily and quickly access the same resources during academic breaks or other disruptions. That is, universities could partner with community resources such as community-based LGBTQ+ centers and spaces, as well as counseling and mental health practitioners. In addition, universities could provide resources for students to learn to identify and navigate LGBTQ+-supportive mental and physical healthcare providers in their own communities, skills that would be useful not only during academic breaks but also as students graduate and transition to more independent living.

## 5. Conclusions

In conclusion, we found that LGBTQ+ students reported increased academic challenges in the first months of the pandemic, including difficulties in managing and concentrating on schoolwork and challenges in balancing their schoolwork with their personal lives. These academic challenges may have far-reaching implications for LGBTQ+ students’ mental health and academic success, particularly given the additional minority-related stressors that LGBTQ+ students often experience, such as family rejection and identity-related stress [15,16]. In addition, these findings underscore the protective role of supportive and quality relationships with family and particularly parents. Identifying factors that protect against increased academic challenges during major life disruptions provides insight to inform universities on how to support students’ academic success year-round.

Our findings contribute to broader discussions on structural barriers affecting LGBTQ+ college students. Future research should explore how these academic challenges compare to challenges faced by other historically marginalized/minoritized student populations and how they manifest across different educational settings, such as online learning. Universities should prioritize inclusive policies, mental health resources, and faculty training, with particular attention on how to reach students during academic breaks and disruptions, to create a more supportive academic environment. By addressing both structural and interpersonal barriers, institutions can better support LGBTQ+ students in achieving academic success, particularly during times of disruption.

## Figures and Tables

**Table 1:** Frequencies for perceived change in academic challenges (*N* = 408).

	Balance School with Personal	Concentrate on Schoolwork	Successfully Manage Tasks
Much easier than before	0.7%	0.7%	2.0%
Somewhat easier than before	1.7%	1.5%	2.7%
A little easier than before	2.5%	5.1%	3.7%
Has not changed	4.9%	7.6%	6.9%
A little harder than before	12.3%	15.4%	17.4%
Somewhat harder than before	19.4%	27.7%	27.0%
Much harder than before	58.6%	41.9%	40.4%

**Table 2:** Linear regression models.

	Perceived Change in Academic Challenges	
Variable	Bivariate β	Multivariate β
Step 1		
Women		0.12
Transgender/GNC		0.14 *
Monosexual		−0.09
Out to parent(s)		0.33 +
Out to others		0.26
Living with parent(s)		−0.04
Living with others		−0.01
Freshman/Sophomore		−0.07
Employed		−0.06
R^2^ (Step 1)		0.05 *
Step 2		
Women		0.10
Transgender/GNC		0.11
Monosexual		−0.09
Out to parent(s)		0.34 *
Out to others		0.27
Living with parent(s)		−0.01
Living with others		0.00
Freshman/Sophomore		−0.04
Employed		−0.06
Social support—family	−0.12 *	−0.04
Social support—friends	0.04	0.06
PARQ	−0.16 **	−0.14 *
R^2^ (Step 2)		0.08 **
R^2^Δ (Step 1–Step 2)		0.03 *

*Note.* + *p* = 0.05, * *p* < 0.05, ** *p* < 0.01. *N* = 408; GNC = gender non-conforming; PARQ = parent–child relationship quality. Bivariate models for social support and relationship quality also included all Step 1 controls.

## Data Availability

We report all data exclusions and all measures used in this paper. We did not conduct manipulations or sample determination analyses. This study was not preregistered. The analysis code and other study materials are available upon reasonable request.
